# Construction of a Near-Infrared Viscosity Fluorescent Probe Targeting Mitochondria and Study on Its pH-Coordinated Effect

**DOI:** 10.3390/molecules31132324

**Published:** 2026-07-02

**Authors:** Xu Tang, Yuxuan Jiang, Yaqin Li, Yunlong Han, Zhi Zhu

**Affiliations:** 1 Institute for Advanced Materials, School of Materials Science and Engineering, Jiangsu University, Zhenjiang 212013, China15298667263@163.com (Y.J.); 15751011724@163.com (Y.L.); tangxu333333@ujs.edu.cn (Y.H.); 2 Institute of the Green Chemistry and Chemical Technology, School of Chemistry and Chemical Engineering Jiangsu University, Zhenjiang 212013, China

**Keywords:** fluorescent probe, viscosity, pH, mitochondria, bio-imaging

## Abstract

In this study, a mitochondria-targeting near-infrared (NIR) fluorescent probe, Mito-V, based on an oxanthrene-like hybrid structure was designed and synthesized, for detecting viscosity with pH-cooperative response characteristics. Upon increasing viscosity, Mito-V exhibits a significant NIR fluorescence enhancement with a high signal-to-noise ratio and excellent selectivity. Under weakly acidic conditions, the viscosity response is further enhanced, demonstrating a pH-cooperative effect that improves detection sensitivity. Cellular imaging experiments confirmed that Mito-V can effectively monitor fluctuations in intracellular viscosity, and co-localization studies revealed its high specificity for mitochondria, with a Pearson’s coefficient of 0.89. Furthermore, the pH-cooperative effect on viscosity detection was verified at the cellular level. In vivo imaging in zebrafish successfully visualized viscosity variations, demonstrating the probe’s applicability and biosafety for monitoring viscosity in biological systems. These findings indicate that Mito-V holds great potential for studying viscosity-related processes in live cells and organisms.

## 1. Introduction

In recent years, the escalating incidence and mortality rates of cancer have posed a significant global public health challenge [1,2,3]. Early diagnosis and precise treatment are pivotal for improving patient survival rates [4,5,6] and the aberrant features of the tumor microenvironment have emerged as novel biomarkers and therapeutic targets for cancer detection [7,8]. Unlike traditional molecular markers (e.g., cell surface receptors, circulating tumor DNA, etc.), the unique physicochemical microenvironment within tumor cells—characterized by abnormal viscosity, low pH, hypoxia, and elevated reactive oxygen species (ROS) levels—has become a promising frontier for cancer diagnosis and therapy [9,10,11,12,13]. Among these features, intracellular viscosity serves as a critical regulator of organelle function and metabolic states, playing a key role in tumor initiation, metastasis, and drug resistance [14,15]. Studies have demonstrated that mitochondrial viscosity is markedly elevated in tumor cells, likely due to dysregulated energy metabolism and oxidative stress responses [16,17,18]. Concurrently, the weakly acidic nature of the tumor microenvironment (pH 6.5–7.0) further exacerbates cellular dysfunction [19,20]. Therefore, the development of molecular probes capable of simultaneously responding to viscosity and pH changes holds great significance for elucidating tumor microenvironment characteristics and advancing precision medicine.

Fluorescence imaging technology has been widely employed for biomarker detection due to its high sensitivity, real-time visualization, and non-invasive nature [21,22,23,24,25,26,27,28,29,30,31,32,33]. Among various imaging tools, near-infrared (NIR) fluorescent probes exhibit immense potential for in vivo imaging owing to their deep tissue penetration and minimal background interference [34,35,36,37]. However, most existing viscosity-sensitive probes suffer from limitations such as insufficient selectivity, low signal-to-noise ratios, or inability to target specific organelles [38,39,40]. Mitochondria, as the cellular powerhouses and apoptosis regulators, exhibit viscosity alterations closely associated with tumor progression [41,42]. Thus, the development of mitochondria-targeted NIR viscosity probes, coupled with an investigation into the pH-dependent synergistic modulation of their detection performance, could significantly enhance the accuracy and specificity of tumor microenvironment monitoring.

This study designed and synthesized a mitochondria-targeting near-infrared viscosity fluorescent probe based on the xanthene scaffold. Systematic characterization confirmed its sensitive viscosity response and pH-dependent properties. The probe’s responsiveness to viscosity was comprehensively analyzed through a series of spectroscopic investigations. The probe utilizes a positively charged oxonium ion structure to achieve mitochondrial targeting through electrostatic attraction between opposite charges, while realizing viscosity-activated fluorescence enhancement via an restriction of intramolecular rotation (RIR) mechanism. In high-viscosity media, the fluorescence intensity of Mito-V exhibits a distinct pH dependence. Under acidic conditions, protonation of the pyronin core enhances the electron-withdrawing capability of the oxonium moiety, thereby strengthening the D–π–A push–pull effect and promoting radiative decay, which consequently leads to an enhancement in fluorescence signal. Consequently, the fluorescence signal can be modulated by microenvironmental acidity, exhibiting higher detection sensitivity under the weakly acidic conditions of tumors. We further evaluated the probe’s selectivity, photostability, and biocompatibility. Additionally, based on cellular imaging, the synergistic effect of a weakly acidic pH on the probe’s viscosity response is discussed. The probe’s viscosity detection capability at the whole-organism level was validated using a zebrafish model. This study not only provides a novel molecular tool for tumor microenvironment research but also offers new insights into the design of multi-parameter-responsive probes.

## 2. Results

### 2.1. Study on the Response Performance of the Probe to Viscosity

**Figure 1:**
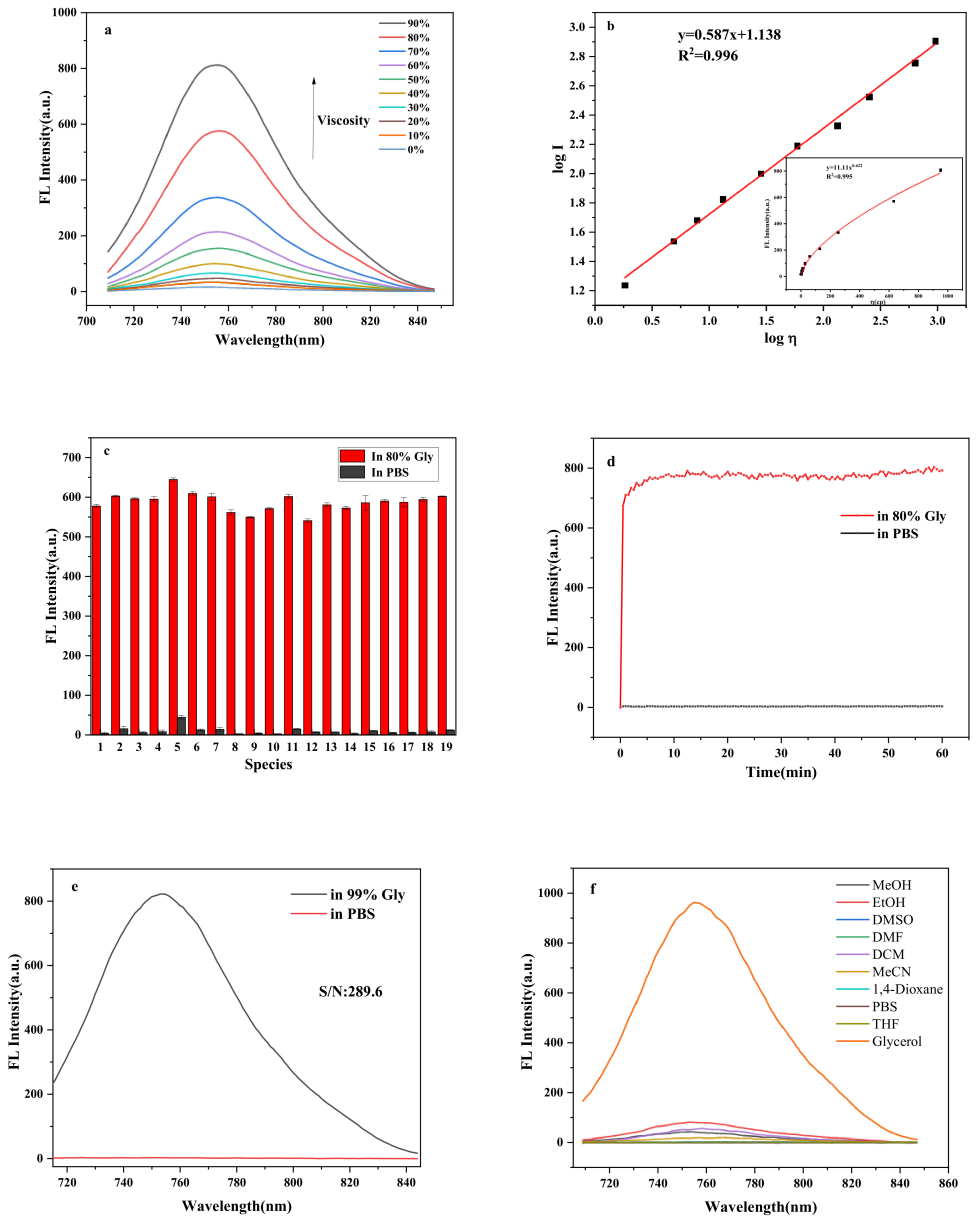
(**a**) Fluorescence spectra of probe Mito-V at different glycerol–methanol ratios; (**b**) fitting curve of log I and log η; (**c**) the fluorescence response of Mito-1 (10 μM) in 80% glycerol–PBS mixture to various interferents. Numbers 1–19 represent H_2_O_2,_ O_2_^−^, OH, Hcy, GSH, Cys, Br^−^, I^−^, F^−^, HS^−^, Ag^+^, Cu^2+^, Fe^3+^, Fe^2+^, Ca^2+^, Mg^2+^, Zn^2+^, Glu, and blank, respectively; (**d**) the fluorescence intensity changes when the Mito-V was added to a system containing 80% glycerol–PBS mixture and to a system with PBS buffer solution; (**e**) signal-to-noise ratio of Mito-V; (**f**) fluorescence spectra of Mito-V in various polar solvents.

To investigate the viscosity sensitivity of the probe Mito-V and evaluate its capability for quantitative viscosity determination in biological systems, the fluorescence spectra of the probe were measured in glycerol–methanol buffer solutions with varying volume ratios, using an excitation wavelength of 700 nm. As shown in Figure 1a, with the increasing proportion of glycerol in the system, the viscosity gradually increased, and the fluorescence emission intensity of the probe at 755 nm enhanced by 47-fold. The fluorescence behavior of the probe can be rationalized by the restriction of the intramolecular rotation (RIR) mechanism [43]. In low-viscosity media, the free rotation of the pendant aryl groups or the diethylamino moieties around the single bonds is facile. Such molecular motions effectively consume the excitation energy through non-radiative decay pathways, leading to a severely quenched fluorescence emission. Upon increasing the viscosity of the microenvironment, the rotational freedom of these moieties is substantially restricted. As a result, the non-radiative relaxation channels are suppressed, and the excited-state energy is preferentially released via radiative decay, thereby giving rise to a pronounced fluorescence turn-on response. In addition, the 4-(diethylamino)phenyl moiety in the probe plays another important role in its photophysical properties. It acts as a strong electron-donating group, which, in combination with the electron-deficient pyronin core, forms an effective D–π–A push–pull system that shifts the emission wavelength into the NIR region. Figure 1b shows the fitted results after taking the logarithm of both viscosity and fluorescence intensity values. A good linear relationship was observed between the logarithm of fluorescence intensity (log I_755_) and the logarithm of viscosity (log η), with the fitting equation y = 0.587x + 1.138 and R^2^ = 0.996. The detection limit for viscosity was calculated to be 1.42 cP based on the equation LOD = 3σ/k, where σ is the standard deviation of the fluorescence intensity at the lowest viscosity point (0% glycerol in PBS, η = 0.89 cP, n = 3) and k is the slope of the linear calibration curve (0.587). The quantum yield of the probe was measured based on the reference method, and the Rh-B was used as the standard substance (Φs = 0.89). Based on the measurement results and formula (Φu = Φs(Yu/Ys)(As/Au)), it is found that the quantum yield of the probe in a 80% volume ratio glycerol system (Φ1) is 0.79, and the quantum yield of probe in a PBS system (Φ) is 0.04. These results indicate that the probe exhibits a strong fluorescence response to viscosity with high sensitivity. Compared with previously reported viscosity probes [44,45,46], the near-infrared excitation and emission characteristics of this probe effectively circumvent the interference from autofluorescence and light scattering in biological specimens, thereby substantially improving the signal-to-noise ratio of detection. Moreover, the probe demonstrates excellent photostability, enabling it to maintain stable fluorescence performance during prolonged imaging and within complex biological environments. These superior attributes collectively endow the probe with broad application prospects for the monitoring and analysis of viscosity in biological systems. In addition, the near-infrared excitation and emission properties of the probe also provide favorable conditions for its subsequent applications in viscosity monitoring and analysis within biological systems.

The anti-interference capability of the probe was further evaluated. The selected probe system consisted of an 80% glycerol–PBS mixed solution, to which various interfering substances, including common cations, anions, reactive oxygen species, and glucose were individually introduced to examine their effects on the probe’s viscosity response. The experimental results demonstrated that the probe Mito-V exhibited strong fluorescence intensity in the high-viscosity 80% glycerol–PBS mixed system. Compared to the blank control group, the presence of interfering substances only caused minor changes in the fluorescence intensity of the probe. Similarly, in low-viscosity probe systems, the interfering substances did not show any significant impact on the fluorescence intensity (Figure 1c).

The response rate and stability of a probe toward viscosity are critical parameters that affect the results of fluorescence imaging experiments. The response rate determines whether the probe can capture dynamic changes in viscosity in real time and accurately, while stability relates to the reliability of its signal output during prolonged imaging processes. This study investigated the changes in fluorescence intensity over time of the probe in both pure PBS buffer and a high-viscosity environment (80% glycerol–PBS mixture). As shown in Figure 1d, upon addition to the high-viscosity system, the probe immediately exhibited a significant increase in fluorescence signal, reached its peak within 5 min, and remained stable thereafter. In contrast, no obvious fluorescence enhancement was observed in the PBS buffer. The results indicate that the probe is capable of rapid response to viscosity and demonstrates excellent photostability. Furthermore, probe Mito-V exhibited a high signal-to-noise ratio in its viscosity response (Figure 1e, SNRa = 289.6), suggesting its suitability for high-contrast near-infrared fluorescence imaging of viscous microenvironments in biological systems. This provides a reliable tool for subsequent applications in live cells or in vivo. Figure 1f shown the ultraviolet–visible absorption spectra and fluorescence spectra of the probe in different solvents, respectively. The ultraviolet absorption peaks of Mito-V are all located near 700 nm in various solvents. Compared with solvents of different polarities, Mito-V exhibits significant fluorescence emission only in highly viscous glycerol, indicating that its fluorescence is minimally affected by polarity. Therefore, in complex biological microenvironments, the fluorescence performance of Mito-V remains largely unaffected by changes in polarity, making it a reliable tool for detecting viscosity in biological systems.

### 2.2. Fluorescence Properties of the Probe in Different pH Systems

**Figure 2:**
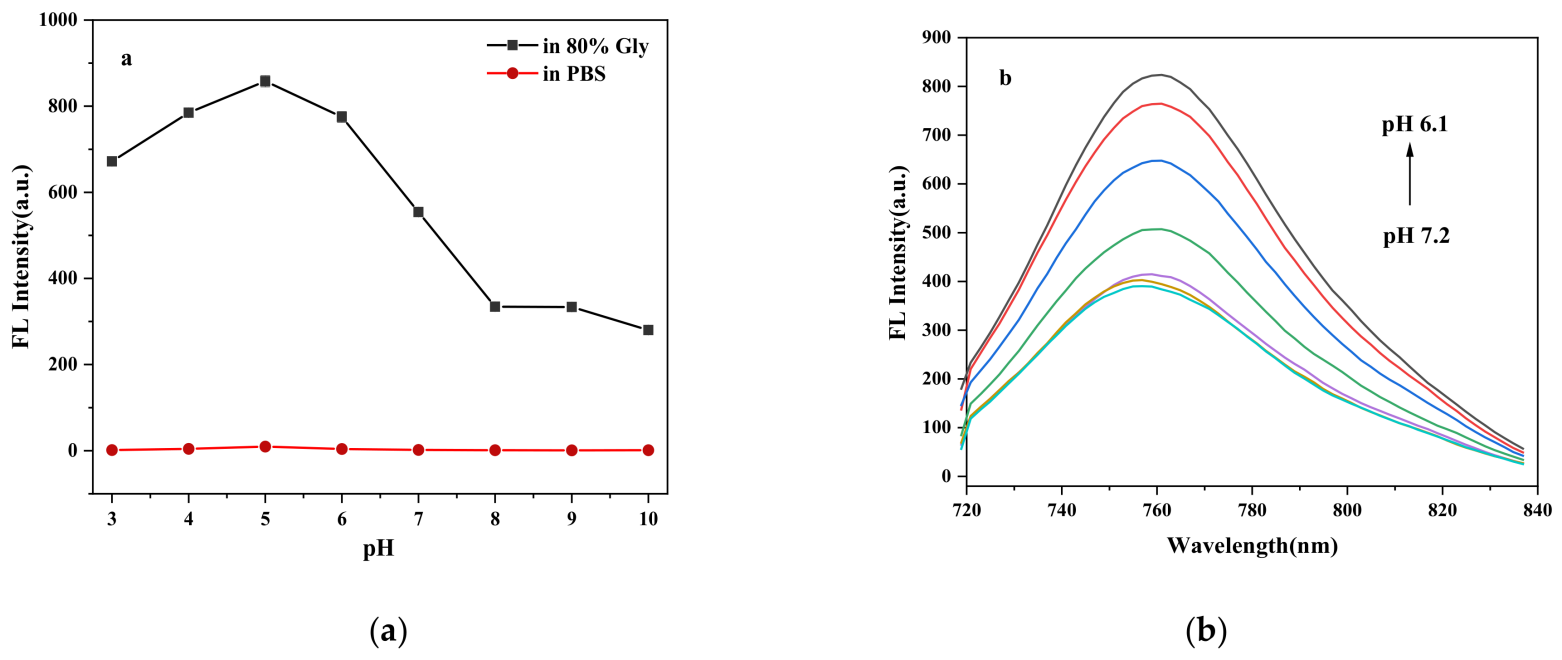
(**a**) Changes in fluorescence intensity of probe Mito-V at different pH values; (**b**) fluorescence response of the probe within the pH range of 6.1–7.2.

To investigate the effect of pH on the viscosity responsiveness of the probe, fluorescence emission spectra were measured both in pure PBS buffer and in a high-viscosity medium containing 80% glycerol at various pH values. As shown in Figure 2a, in the high-viscosity 80% glycerol system, the fluorescence intensity of Mito-V exhibits a distinct pH-dependent profile, reaching a maximum at pH 5 (~850), while decreasing sharply under neutral-to-alkaline conditions (pH 7–10). In contrast, in PBS buffer (low viscosity), the fluorescence intensity remains nearly constant (~330–355) across the pH range of 6.1–7.2 (Figure 2b), indicating that pH alone exerts negligible regulatory effects on the emission in low-viscosity media. This phenomenon can be rationalized by the synergistic interplay between ICT enhancement and RIR restriction. Under acidic conditions, protonation of the pyronin core increases the electron-withdrawing capability of the oxonium moiety, thereby strengthening the D–π–A push–pull effect and promoting radiative decay. However, in low-viscosity media, the rapid intramolecular rotation of the 4-(diethylamino)phenyl moiety still mediates efficient non-radiative relaxation, which largely counteracts the ICT-induced fluorescence enhancement. In high-viscosity media (80% glycerol), the same rotational motion is physically constrained via the RIR mechanism, effectively suppressing non-radiative decay. Consequently, the increased radiative rate arising from ICT and the suppressed non-radiative rate arising from RIR act synergistically, such that a pronounced fluorescence enhancement is achieved only when both acidic and high-viscosity conditions are satisfied simultaneously. pH and viscosity exert synergistic modulatory effects on the fluorescence response of Mito-V, resulting in higher sensitivity of the probe to viscosity under weakly acidic conditions. This characteristic provides a favorable basis for efficient monitoring of viscosity in the weakly acidic microenvironment of tumor cells.

### 2.3. Cytotoxicity of the Probe

**Figure 3:**
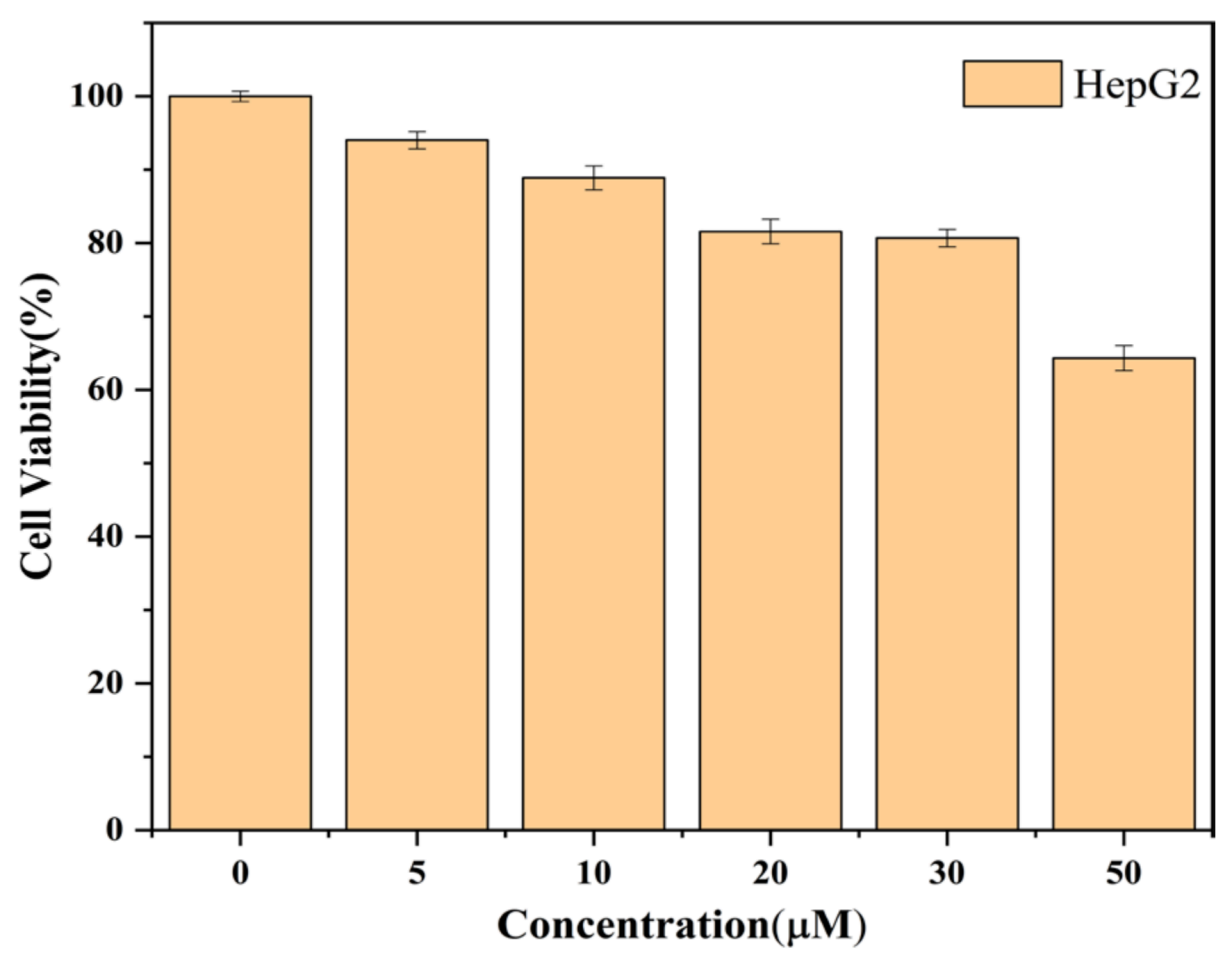
The relationship between the cell viability of HepG2 and the concentration of Mito-V.

To assess the biosafety of the Mito-V probe, we evaluated its cytotoxicity using the CCK-8 assay and other experimental methods. As shown in Figure 3, even when the probe concentration reached 30 μM, the cell viability remained above 80% and the IC_50_ value was significantly higher than this concentration. This confirms that Mito-V exhibits negligible cytotoxicity within this concentration range. Considering its excellent viscosity-sensitive response (as mentioned previously) and low cytotoxicity, this study determined 10 μM to be the optimal working concentration for subsequent cell imaging experiments.

### 2.4. Co-Localization Experiments

**Figure 4:**
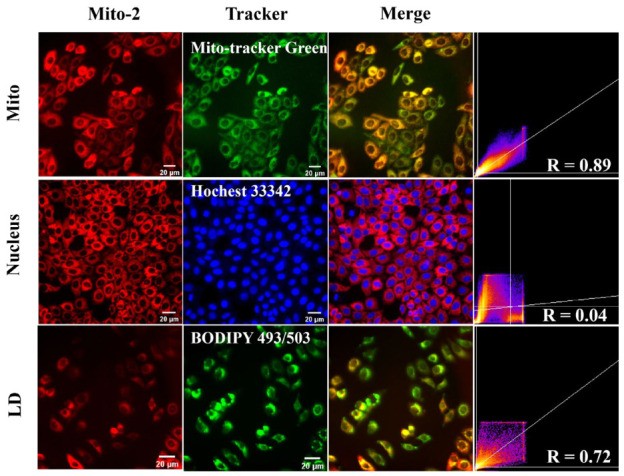
Co-localization of probe Mito-1 with commercial dyes for mitochondria, nucleus, and lipid droplets in HepG2.

Given that the structure of Mito-V contains a positively charged oxonium ion group, it is expected to target mitochondria through electrostatic interaction with the negatively charged mitochondrial membrane. To evaluate the mitochondrial targeting capability of Mito-V, co-localization imaging was performed using Mito-V together with MitoTracker Green. Since Mito-V emits in the near-infrared region (displayed as red fluorescence), the commercial mitochondrial dye MitoTracker Green (green fluorescence) was chosen as the reference marker. As shown in Figure 4, the red fluorescence channel of Mito-V overlapped well with the green fluorescence channel of MitoTracker Green in HepG2 cells, yielding a Pearson’s correlation coefficient (PCC) of 0.89. In contrast, Mito-V exhibited poor co-localization with both the nucleus and lipid droplets, with PCC values of 0.04 and 0.72, respectively. These results demonstrate that Mito-V can specifically target mitochondria in living cells.

### 2.5. Imaging of Starvation-Induced Intracellular Viscosity Changes with the Probe

**Figure 5:**
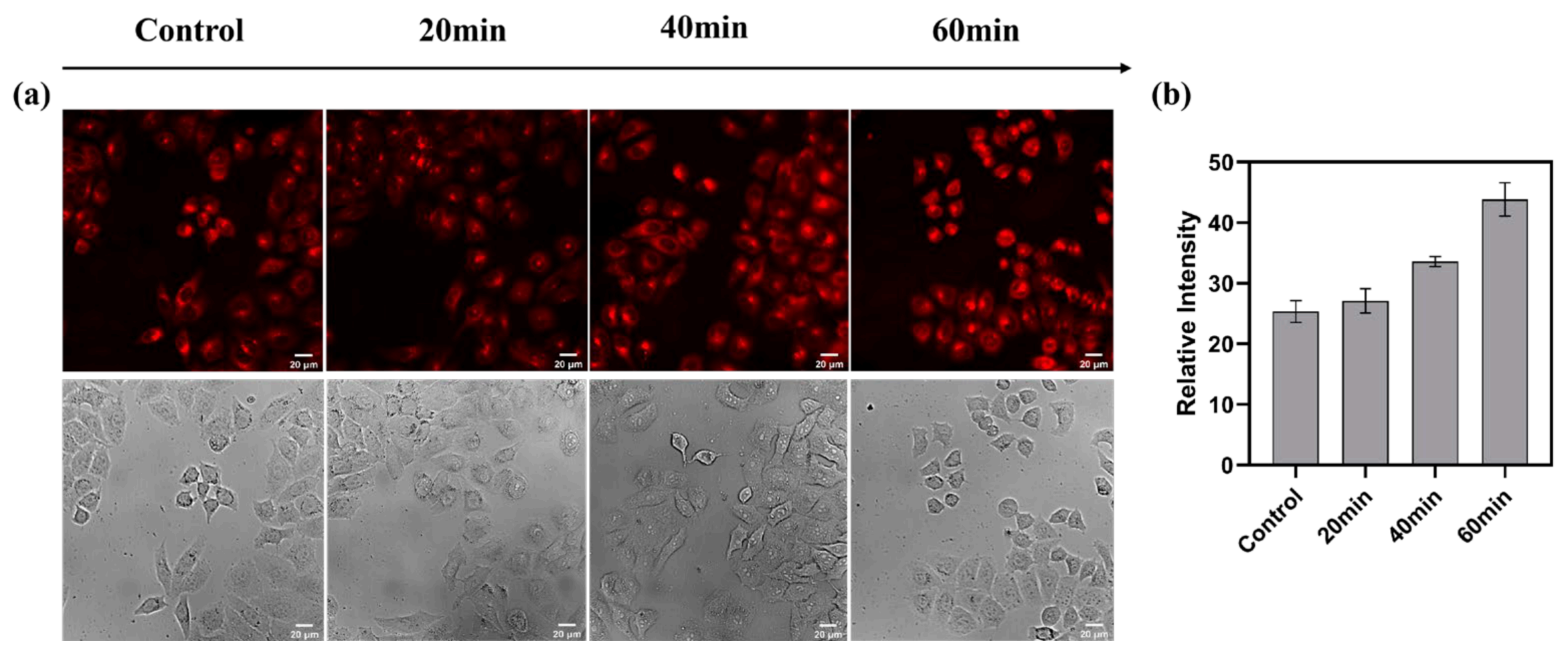
(**a**) Fluorescence imaging of HepG2 co-cultured with Mito-V after different starvation times; (**b**) fluorescence intensity in the red channel.

The viscosity imaging results of HepG2 cells under starvation conditions are shown in Figure 5. Within the initial 20 min after treatment with FBS-free medium, the relative fluorescence intensity of the cells increased only modestly, likely due to insufficiently pronounced metabolic alterations and viscosity changes at this early stage. However, after 40 and 60 min of starvation treatment, a notable enhancement in fluorescence intensity was observed compared to the control group, with a relative increase of approximately 1.7-fold. This phenomenon can be attributed to the accumulation of metabolic waste products—such as lactic acid and fatty acids—resulting from elevated metabolic activity under nutrient deprivation. These substances contribute to an increase in intracellular viscosity, thereby leading to the enhanced fluorescence signal.

### 2.6. Imaging of Drug-Induced Intracellular Viscosity Changes with the Probe

**Figure 6:**
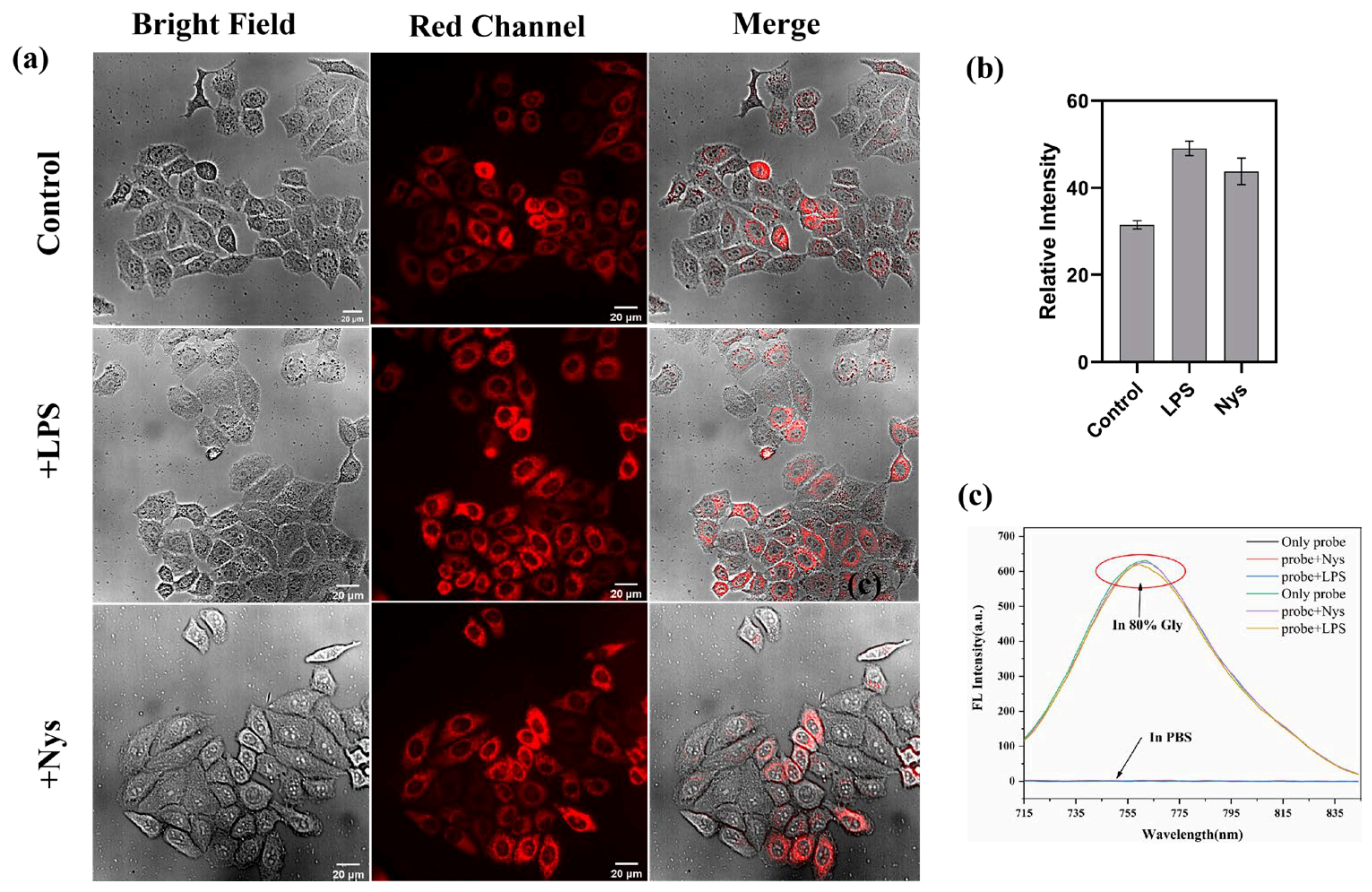
(**a**) Fluorescence imaging of HepG2 co-cultured with LPS and Nys, followed by co-culture with Mito-V; (**b**) fluorescence intensity of the red channel corresponding to (**a**); (**c**) fluorescence emission spectra of the probe after adding LPS and Nys in a PBS buffer system and 80% glycerol–PBS mixture system.

As shown in Figure 6a,b, the imaging results of Mito-V for drug-induced intracellular viscosity changes revealed a noticeable increase in mitochondrial viscosity upon stimulation with LPS or nystatin (Nys), compared to the control group. The potential influence of LPS and Nys on the intrinsic fluorescence properties of Mito-V was evaluated in both PBS buffer and an 80% glycerol–PBS mixture. The results, presented in Figure 6c, demonstrated that the presence of LPS or Nys had only a negligible effect on the fluorescence performance of Mito-V. This confirms that the enhanced fluorescence intensity observed in cellular imaging is indeed due to alterations in intracellular viscosity levels induced by LPS and Nys in HepG2 cells. These findings collectively demonstrate that Mito-V can be successfully applied for monitoring viscosity changes in living cells.

### 2.7. Imaging of Intracellular Viscosity Changes Under Different pH Environments Using the Probe

**Figure 7:**
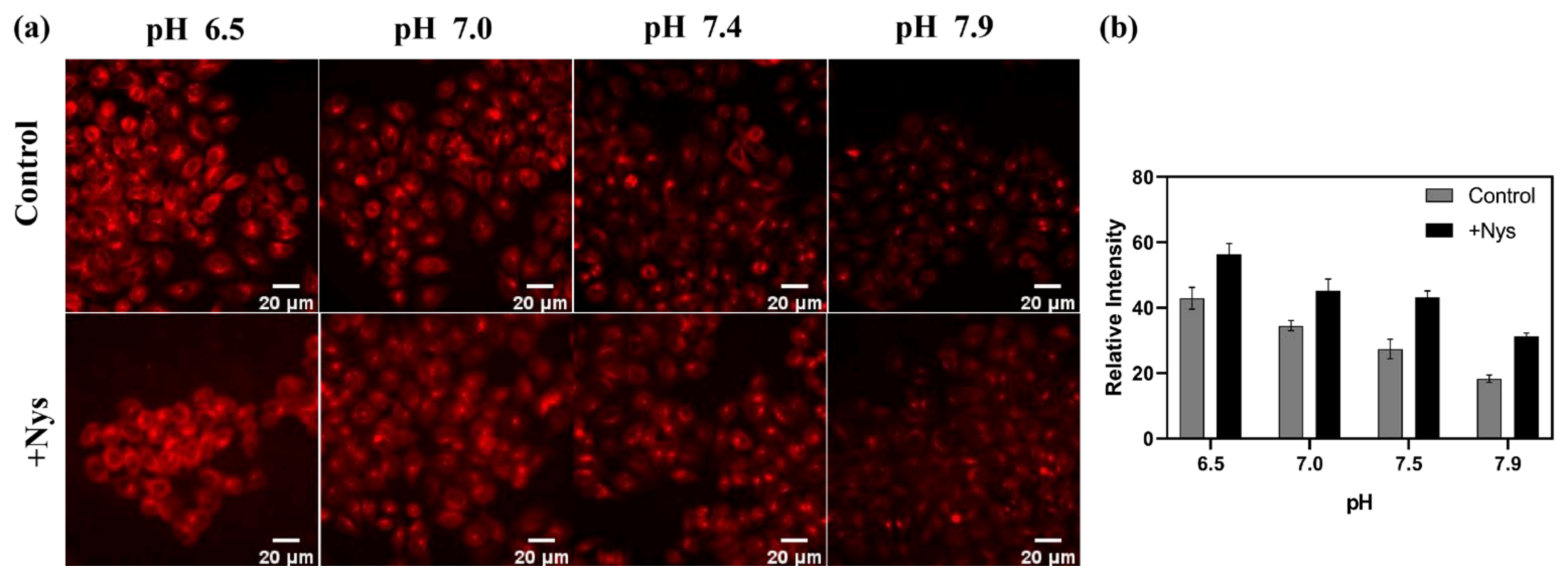
(**a**) Fluorescence imaging of HepG2 cells before and after stimulation, treated with different pH levels; (**b**) relative fluorescence intensity in the red channel.

Spectroscopic experiments have confirmed that a weakly acidic environment exhibits a synergistic effect on the viscosity response of the probe Mito-V. Based on this finding, we further investigated the performance of the probe in imaging intracellular viscosity under different pH conditions (6.5, 7.0, 7.4, and 7.9). As shown in Figure 7, the fluorescence signals in cellular imaging gradually increased as the pH decreased. Furthermore, when cells under various pH conditions were stimulated with drugs to induce elevated viscosity levels, the fluorescence intensity increased across all pH groups compared to untreated cells. More importantly, the pH-dependent trend in fluorescence enhancement was consistent with the observations from spectroscopic studies, reaffirming that an acidic pH synergistically enhances the viscosity response of the probe.

### 2.8. Zebrafish Imaging

**Figure 8:**
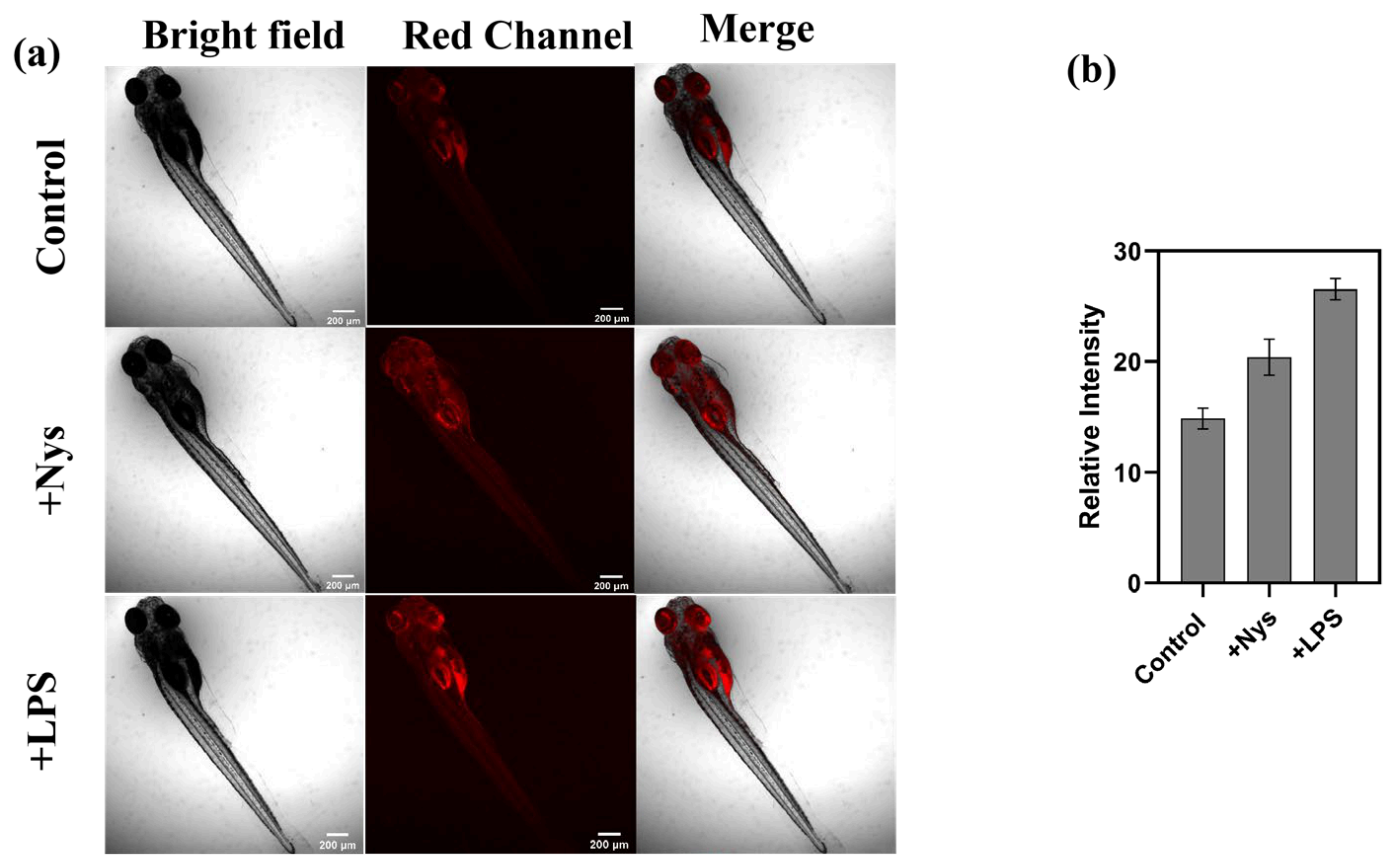
(**a**) Fluorescence imaging of zebrafish untreated and treated with Nys and LPS co-cultured with Mito-V; (**b**) relative fluorescence intensities in the red channel.

Based on the above experiments, we have demonstrated that Mito-V can monitor viscosity changes in the mitochondria of HepG2 cells. Owing to its emission in the near-infrared region, Mito-V exhibits a strong capability for deep-tissue imaging. We further investigated its responsiveness to viscosity and its performance in live-cell imaging within zebrafish. As shown in Figure 8, zebrafish treated with Mito-V alone exhibited weak fluorescence. In contrast, a significant enhancement of red-channel fluorescence was observed in zebrafish co-treated with lipopolysaccharide and nystatin followed by Mito-V staining, further confirming the probe’s utility for in vivo imaging. These results collectively demonstrate that Mito-V can effectively monitor viscosity changes in live zebrafish through fluorescence imaging.

## 3. Materials and Methods

### 3.1. Chemicals and Instrumentations

All chemicals are from commercial suppliers ((Bidepharm.com, Shanghai, China; and Aladdin, Shanghai, China). All experimental water was purified by secondary distillation. The fluorescence emission spectra were recorded by an Agilent Cary Eclipse G9800 A fluorescence spectrophotometer (Agilent Technologies, Santa Clara, CA, USA). The fluorescence image was obtained by a Leica TCS SP5 laser confocal microscope (Agilent Technologies, Santa Clara, CA, USA). The UV-vis absorption spectra were measured by using a Cary 8454 spectrophotometer (Agilent Technologies, Santa Clara, CA, USA). Cell viability was measured using a Synergy H1 microplate reader (BioTek Instruments, Winooski, VT, USA). The pH was determined by an Orion-star A211 pH meter (Thermo Fisher Scientific, Waltham, MA, USA). The NMR spectra were recorded on a Bruker DRX-400 spectrometer (Bruker Corporation, Billerica, MA, USA). Mass spectrometry was performed using an Q-Exactive spectrometer (Thermo Fisher Scientific, Waltham, MA, USA).

### 3.2. Synthesis of Probe Mito-V

Compound **3-1**: A round-bottom flask was charged with 25 mL of concentrated H_2_SO_4_ and cooled in an ice-water bath to maintain the temperature at 0–5 °C. Cyclohexanone (1.47 g, 0.015 mol) was then added dropwise under stirring. After the mixture was warmed to room temperature, 4-(diethylamino)salicylaldehyde (1.93 g, 0.02 mol) was slowly introduced. The reaction mixture was heated to reflux at 90 °C in an oil bath with vigorous stirring for 3.0 h. Upon completion, the mixture was cooled to room temperature and poured into 200 mL of ice water. Subsequently, 1.8 mL of 70% perchloric acid was added, and the solution was allowed to stand overnight, yielding a red precipitate. The solid was collected by filtration, washed with cold deionized water (100 mL × 3), and dried to afford 2.525 g of the target compound **3-1** as a red solid in 71% yield. ^1^H NMR (400 MHz, CDCl_3_) δ 8.43 (s, 1H, Ar-H), 7.94 (d, *J* = 9.6 Hz, 1H, Ar-H), 7.28 (dd, *J* = 9.5, 2.6 Hz, 1H, Ar-H), 6.82 (d, *J =* 2.4 Hz, 1H, Ar-H), 3.66 (q, *J =* 7.2 Hz, 4H, NCH_2_CH_3_), 3.04 (t, *J =* 6.4 Hz, 2H, CH_2_-cyclohexene), 2.85 (t, *J =* 6.2 Hz, 2H, CH_2_-cyclohexene), 2.05–1.84 (m, 4H, CH_2_-cyclohexene), 1.33 (t, *J =* 7.2 Hz, 6H, NCH_2_CH_3_). ^13^C NMR (101 MHz, CDCl_3_) δ 170.54, 159.83, 156.32, 150.38, 132.89, 122.18, 119.40, 118.48, 95.06, 77.16, 76.84, 76.53, 46.25, 28.94, 26.04, 21.19, 21.00. HRMS (ESI): *m*/*z* calculated for [C_17_H_22_ON ^+^ ]: 256.17, found: 256.18.

**Scheme 1:**
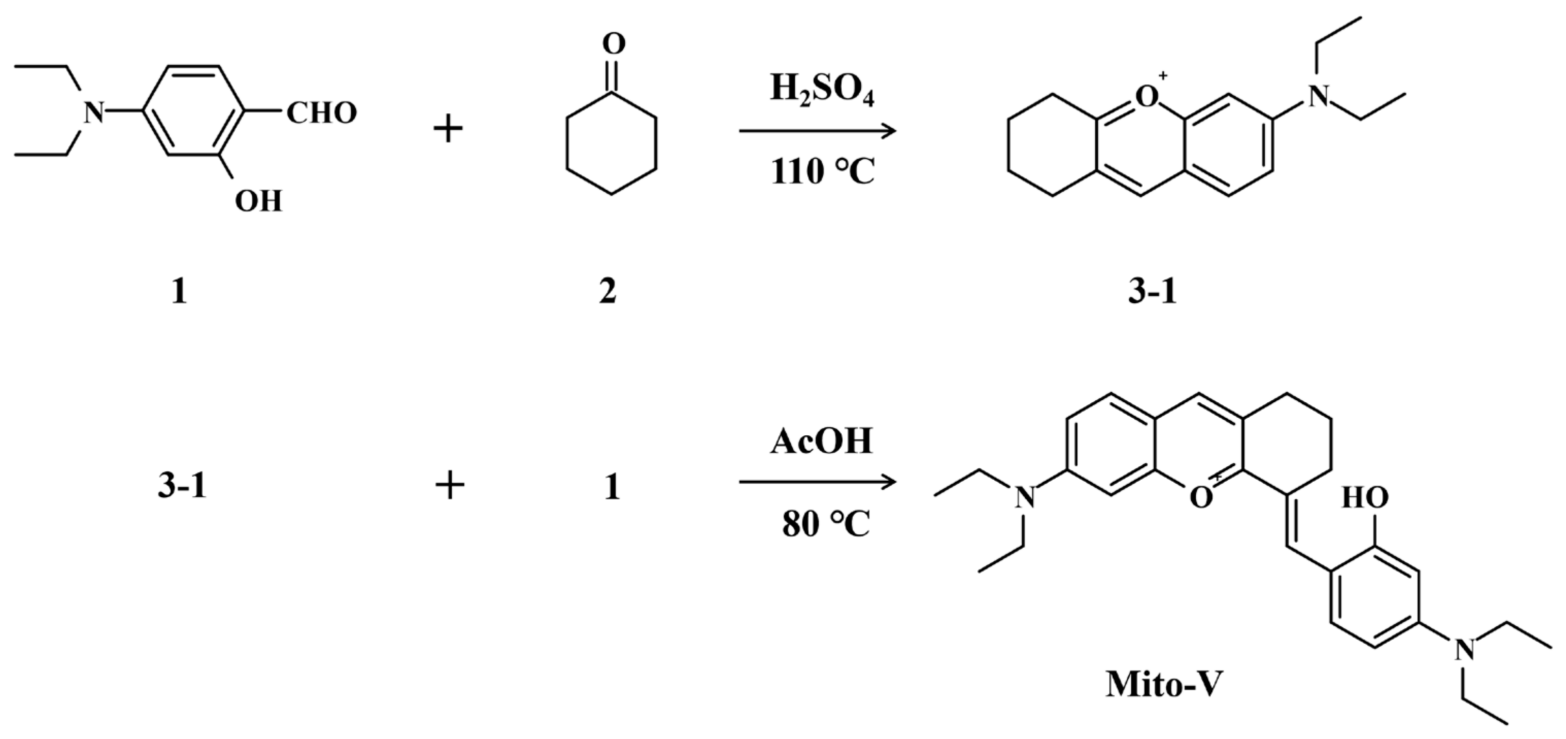
Schematic diagram of the synthesis route for probe Mito-V.

Probe Mito-V: Mito-V was synthesized via the condensation of 4-(diethylamino)salicylaldehyde (0.965 g, 5 mmol) and compound **3-1** (1.28 g, 5 mmol) in 30 mL of glacial acetic acid. The reaction mixture was heated under reflux at 110 °C for 3 h. After completion, the solvent was removed under reduced pressure using a rotary evaporator. The crude product was further purified by column chromatography using a dichloromethane/methanol (20:1, *v*/*v*) eluent system, yielding the desired product as a blue solid (49% yield). ^1^H NMR (400 MHz, DMSO-d_6_) δ 10.43 (s, 1H, OH), 8.36 (s, 1H, =CH-bridge), 8.15 (s, 1H, Ar-H), 7.71 (d, *J =* 9.3 Hz, 1H, Ar-H), 7.47 (d, *J =* 9.2 Hz, 1H, Ar-H), 7.20 (dd, *J =* 9.3, 2.4 Hz, 1H, Ar-H), 6.90 (d, *J =* 2.4 Hz, 1H, Ar-H), 6.37 (dd, *J =* 9.3, 2.6 Hz, 1H, Ar-H), 6.25 (d, *J =* 2.6 Hz, 1H, Ar-H), 3.62 (q, *J =* 7.1 Hz, 4H, NCH_2_CH_3_), 3.48–3.38 (m, 4H, NCH_2_CH_3_), 2.83 (dt, *J =* 28.2, 6.1 Hz, 4H, CH_2_-C=C), 1.83 (t, *J =* 6.1 Hz, 4H, β-CH_2_ CH_2_-C=C), 1.21 (t, *J =* 7.0 Hz, 12H, NCH_2_CH_3_). ^13^C NMR (101 MHz, DMSO-d6) δ 164.74, 161.00, 157.13, 153.63, 151.99, 144.43, 135.43, 132.42, 130.66, 122.49, 119.46, 115.39, 115.26, 112.12, 105.10, 96.38, 94.95, 55.77, 44.69, 44.05, 43.86, 39.94, 39.73, 39.52, 39.31, 39.10, 38.89, 38.68, 27.43, 27.07, 20.87, 18.30, 12.44, 12.20. HRMS(ESI): *m*/*z* calculated for [C_28_H_35_O_2_N_2_^+^] = 431.27, found [C_28_H_35_O_2_N_2_^+^] = 431.34. (Scheme 1 and Figures S1–S6).

### 3.3. Optical Property Evaluation

Viscosity response: The fluorescence emission spectra of the probe were measured in systems of varying viscosity (glycerol/PBS mixtures) at a probe concentration of 10 μM. Additionally, the fluorescence emission spectra were recorded in the presence of different interferents, each at a concentration of 100 μM, with the probe concentration maintained at 10 μM. The excitation wavelength was set at 700 nm for all measurements [47].

pH-dependent fluorescence response: The fluorescence emission spectra of the probe were acquired in PBS buffer solutions and in 80% glycerol–PBS mixed solvent systems across a pH range of 3 to 10, at a probe concentration of 10 μM. The emission spectra were collected within a narrower pH range of 6.2 to 7.4, at intervals, in the 80% glycerol–PBS mixed solvent system.

Solvatochromism assessment: The probe was dissolved in solvents of varying polarity to form test solutions with a concentration of 10 μM. The ultraviolet–visible (UV-Vis) absorption and fluorescence emission spectra of these solutions were subsequently recorded.

### 3.4. Applications in Biological Systems

#### 3.4.1. Cytotoxicity Assay

The cytotoxicity and biocompatibility of the probe Mito-V were evaluated using the CCK-8 assay. The HepG2 cells were selected for this experiment. The cell culture medium consisted of DMEM supplemented with 10% fetal bovine serum and 1% penicillin–streptomycin [48]. The HepG2 cells were seeded in a 96-well plate at a density of 1.0 × 10^4^ cells per well and cultured for 24 h. Subsequently, Mito-V solutions with varying concentration gradients were added to the wells. After an additional 24 h of incubation, 10 μL of CCK-8 solution was added to each well, followed by 2 h of incubation. The optical density (OD) at 450 nm was measured using a microplate reader, and the cell viability rate was calculated based on the prescribed formula.

#### 3.4.2. Co-Localization Imaging

Co-localization imaging experiments were performed using the commercial mitochondrial dye MitoTracker Green and the probe Mito-V to validate the mitochondrial targeting specificity of Mito-V. Concurrently, rapid organelle co-localization imaging was conducted under a fluorescence microscope using the lipid droplet-specific dye BODIPY493/503 and the nuclear stain Hoechst 33342. The distribution of the probe within various organelles was quantitatively assessed based on Pearson’s co-localization coefficient [49].

#### 3.4.3. Imaging of Viscosity Changes Induced by Starvation

Under nutrient-deficient conditions, i.e., starvation, cells are unable to carry out normal metabolic activities, leading to the accumulation of metabolic products and a consequent increase in intracellular viscosity. Additionally, starvation can induce enhanced autophagic activity, which also contributes to elevated cytoplasmic viscosity [50,51]. Therefore, investigating starvation-induced changes in intracellular viscosity is essential for understanding cellular responses to nutritional stress.

In this experiment, adherent HepG2 cells were subjected to starvation by culturing in fetal bovine serum (FBS)-free medium for 20, 40, and 60 min. Subsequently, 10 µL of the probe solution was added to each well, resulting in a final probe concentration of 10 µM. The cells were incubated with the probe for 30 min, washed three times with PBS, and immediately subjected to live-cell imaging.

#### 3.4.4. Imaging of Intracellular Viscosity Changes Induced by Nystatin

To validate the capability of the probe to monitor changes in intracellular viscosity, nystatin was employed to stimulate cells and alter their internal viscosity. Nystatin is a polyene antifungal antibiotic that binds to sterols in the cell membrane, compromising its integrity and increasing membrane permeability. This effect leads to the leakage of intracellular components, which may result in changes in the intracellular environment, including an increase in viscosity [52]. In this experiment, HepG2 cells were co-incubated with nystatin (10 μM) for different durations (20, 40, and 60 min) to induce increased intracellular viscosity, with a control group included for comparison. Subsequently, the probe (10 μM) was added to the cells and incubated for 30 min, followed by three washes with PBS before fluorescence imaging was performed.

#### 3.4.5. Cellular Imaging Under Different pH Conditions

The imaging of cells under varying pH conditions was conducted in two separate experimental groups. In the first group, HepG2 cells were pretreated with PBS buffers at different pH levels for 30 min, followed by the addition of the probe Mito-V. The probe was then co-incubated with the cells for 30 min. After incubation, the cells were washed three times with PBS before fluorescence imaging was performed. In the second group, HepG2 cells were first stimulated with the drug LPS for 30 min and subsequently cultured in environments with different pH levels.

#### 3.4.6. Zebrafish Imaging Experiment

Zebrafish were placed in a 24-well plate with 1 mL of culture medium per well. They were pretreated with oleic acid (OA) and nystatin (Nys) for 30 min, followed by the addition of 10 μL of probe stock solution (1 mM) to achieve a final concentration of 10 μM. After an additional 30 min of incubation, the stained zebrafish were carefully transferred using a rubber-bulb pipette and washed with PBS to remove surface-adsorbed probe. For fluorescence imaging, zebrafish were anesthetized for 15 s and then placed on a clean glass slide. Viscosity levels in zebrafish treated with nystatin and lipopolysaccharide were monitored using fluorescence microscopy.

## 4. Conclusions

Based on the restriction of the intramolecular rotation (RIR) mechanism, we designed and synthesized a viscosity-responsive probe with an extended conjugate structure that operates in the near-infrared (NIR) region for both excitation and emission. Weakly acidic conditions were found to synergistically enhance the viscosity response, significantly improving the sensitivity of the probe. Comprehensive spectroscopic characterization demonstrated that Mito-V exhibits a high signal-to-noise ratio (289.6-fold) and excellent selectivity against potential interferents. The probe showed low cytotoxicity and was capable of specifically localizing to mitochondria. It was successfully applied for imaging viscosity changes both in live cells and in vivo in zebrafish. Moreover, the synergistic effect of acidic pH on viscosity sensing was further verified during cellular imaging. The NIR-excitable and NI-emissive properties of Mito-V, coupled with its pH-assisted response to viscosity, provide strong support for its potential biological applications.

## Data Availability

Data are contained within the article and Supplementary Materials.

## Supplementary Materials

The following supporting information can be downloaded at https://www.mdpi.com/article/10.3390/molecules31132324/s1, Figure S1: 1H NMR (400 MHz, CDCl3) spectrum of compound 3-1; Figure S2: ^13^C NMR (101MHz, CDCl_3_) spectrum of compound 3-1; Figure S3: HRMS (ESI) spectrum of compound 3-1; Figure S4: ^1^H NMR (400 MHz, DMSO-d6) spectrum of Mito-V; Figure S5: 13C NMR (101 MHz, DMSO-d6) spectrum of Mito-V; Figure S6: HRMS (ESI) spectrum of Mito-V.
