# Supplementary material for: Construction of a Near-Infrared Viscosity Fluorescent Probe Targeting Mitochondria and Study on Its pH-Coordinated Effect
Source: Molecules. 2026 Jul 2;31(13):2324. doi: 10.3390/molecules31132324 (PMC13362614; doi:10.3390/molecules31132324)
Supplement: Supplementary file 1 [file molecules-31-02324-s001.zip › molecules-4390046-supplementary.pdf]

Construction of a Near-Infrared Viscosity Fluorescent Probe Targeting Mitochondria and Study on Its pH-Coordinated Effect

Xu Tang[a*], Yuxuan Jiang[a], Yaqin Li[a], Yunlong Han[a], Zhi Zhu[b *]

[a] Institute for Advanced Materials, School of Materials Science and Engineering, Jiangsu university, Zhenjiang, Jiangsu, 212013, China.

[b] Institute of the Green Chemistry and Chemical Technology, School of Chemistry and Chemical

Engineering, Jiangsu university, Zhenjiang, Jiangsu, 212013, China.

Corresponding author: Xu Tang, E-mail: tangxu@ujs.edu.cn; Zhi Zhu, E-mail: zhuzhi@ujs.edu.cn;

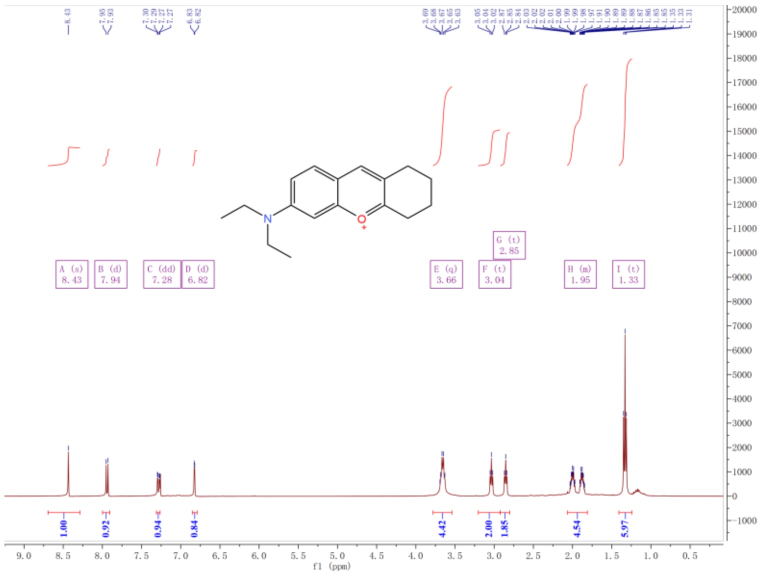

Figure S1 ^{1}H NMR (400 MHz, CDCl3) spectrum of compound 3-1.

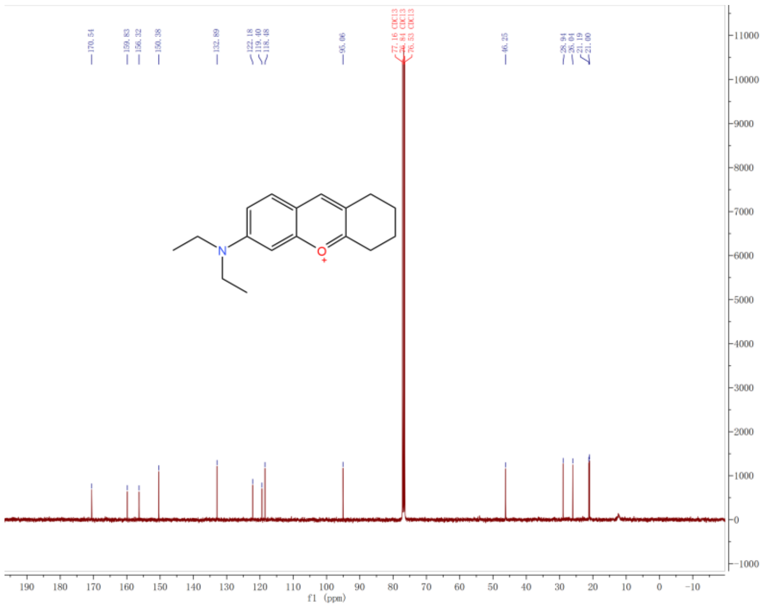

Figure S2 ^{13}C NMR (101MHz, $CDCl_3$) spectrum of compound 3-1.

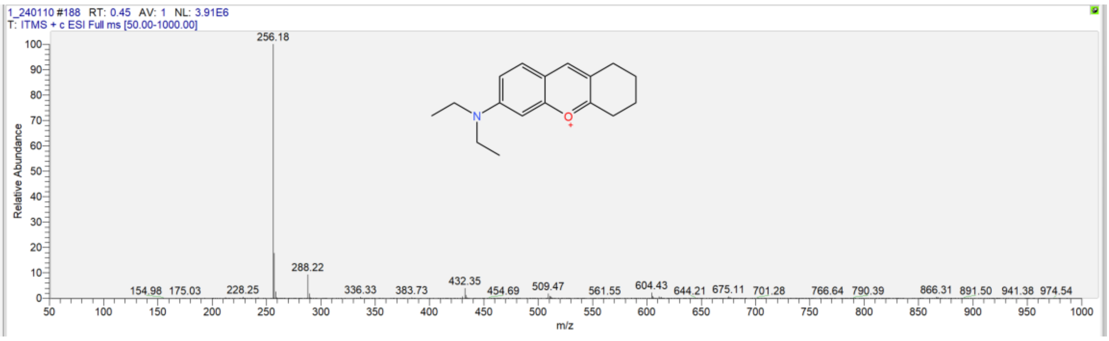

Figure S3 HRMS (ESI) spectrum of compound 3-1.

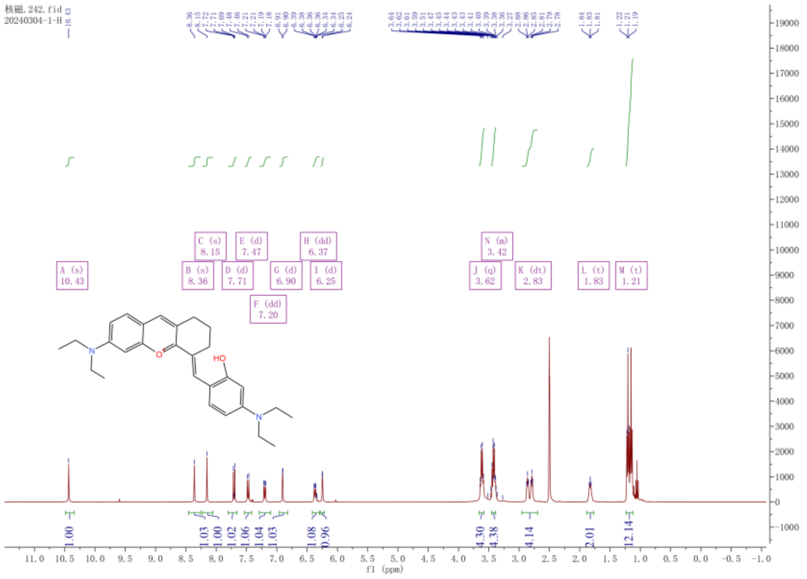

Figure S4 ^{1}H NMR (400 MHz， DMSO-d6) spectrum of Mito-V.

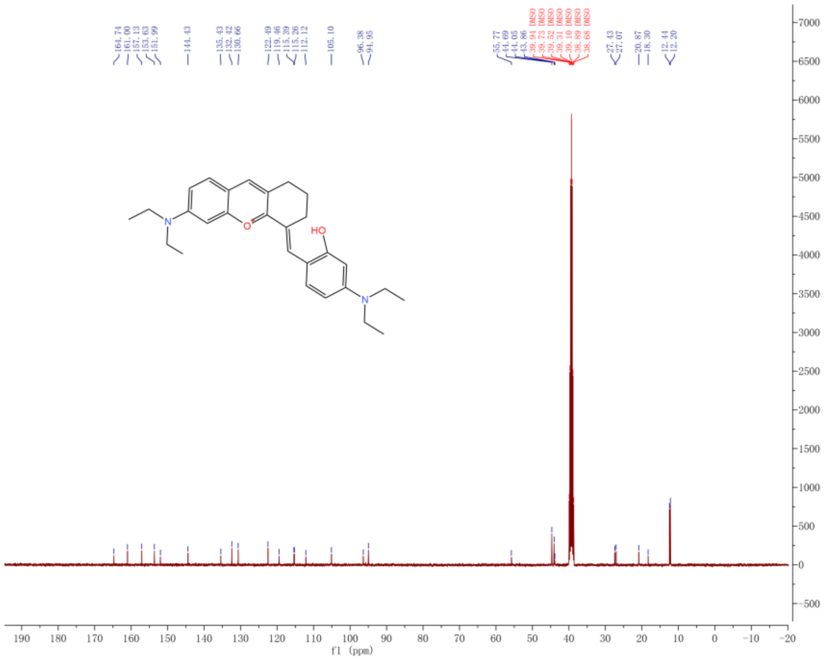

Figure S5 ^{13}C NMR (101 MHz， DMSO-d6) spectrum of Mito-V.

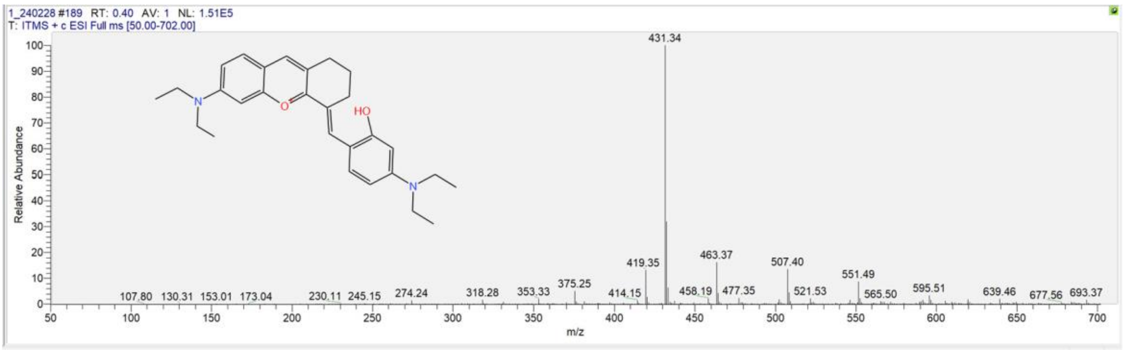

Figure S6 HRMS (ESI) spectrum of Mito-V.
